# The complete chloroplast genome sequence of *Clivia robusta*

**DOI:** 10.1080/23802359.2021.2017370

**Published:** 2022-01-05

**Authors:** Xing-Hua Zhao, Ling Yue, Xiu-Li Feng, Dan Li, Hai-Hong Wu, Xu-Hui Chen

**Affiliations:** a Institute of Floriculture, Liaoning Academy of Agricultural Sciences, Shenyang, China; b College of Bioscience and Biotechnology, Shenyang Agricultural University, Shenyang, China

**Keywords:** *Clivia robusta*, chloroplast genome sequence, phylogenetic tree

## Abstract

In this study, we reported the complete chloroplast genome sequence of *Clivia robusta* for the first time. The complete chloroplast genome of *C. robusta* was 157,130 bp in length, containing a large single-copy region (LSC, 85,430 bp), a small single-copy region (SSC, 18,278 bp), and two inverted repeat regions (IRs, 26,711 bp). The overall GC content was 38.01%. The chloroplast genome contained 128 genes in total, including 86 protein-coding, 34 tRNA, and eight rRNA genes. The phylogenetic tree showed that *C. robusta* formed a monophyletic clade with other *Clivia* species.

*Clivia robusta* B.G. Murray 2004 belongs to genus *Clivia* in the family Amaryllidaceae. It is an evergreen herbaceous plant, flowering from late March to early August. Its flowers are pendulous and tubular, orange with green tips (Murray et al. 2004). Due to its high ornamental value, we sequenced and analyzed the complete chloroplast genome of *C. robusta* for the first time, in order to better understand its relationship with related species and provide a scientific basis for hybridization and breeding.

Young fresh leaves of *C. robusta* were sampled from Liaoning Academy of Agricultural Sciences (N41°48’33”, E123°34’53”), Shenyang, China. Total genomic DNA was isolated following the modified CTAB method for constructing a 400 bp shotgun library, and a specimen was deposited at the Institute of Floriculture of Liaoning Academy of Agricultural Sciences (www.laas.cn, contact person XH Zhao, and email zhaohaihong1997@163.com) under the voucher number ZZJZL03. The complete chloroplast genome of *C. robusta* was sequenced on the Illumina NovaSeq platform, assembled with GetOrganelle v1.6.2e (Jin et al. 2020), and annotated with the OGAP pipeline (https://github.com/zhangrengang/OGAP). The draft annotations were then adjusted manually. The annotated complete chloroplast genome of *C. robusta* was deposited in GenBank with accession number MW660367. Phylogenetic tree was generated by maximum likelihood (ML) analysis. The complete chloroplast genomes of 23 representative species of the family Amaryllidaceae were selected, using *Asparagus officinalis* (Asparagaceae) as an outgroup. The sequences were aligned using MAFFT v7.471 (Standley and Katoh 2013), and the multiple alignment was trimmed with trimAl v1.2 (Capella-Gutierrez et al. 2009). The phylogenetic tree was reconstructed with the software IQ-TREE v1.6.5 (Nguyen et al. 2015). The best-fit K3Pu + F+R3 model was chosen according to the Bayesian information criterion (BIC) using ModelFinder (Kalyaanamoorthy et al. 2017). Ultrafast bootstrap (Hoang et al. 2018) was used to test branch support values with 1000 replicates.

The complete chloroplast genome of *C. robusta* was 157,130 bp in length and displayed a typical quadripartite structure, which contained a pair of inverted repeat regions (IRs, 26,711 bp) separated by a large single-copy region (LSC, 85,430 bp) and a small single-copy region (SSC, 18,278 bp). The overall GC content was 38.01%. The chloroplast genome of *C. robusta* contained 128 genes, including 86 protein-coding, 34 tRNA, and eight rRNA genes. There were 19 genes duplicated in the IR regions, including 7 protein-coding genes, eight tRNA, and four rRNA genes. The phylogenetic tree showed that *C. robusta* and other *Clivia* species formed a monophyletic clade within the family Amaryllidaceae with 100% bootstrap values (Figure 1). The degrees of differentiation between *C*. *robusta* and other *Clivia* species (*C. caulescens, C. gardenii, C. miniata,* and *C. miniata* var*. citrina*) were 0.41%, 0.44%, 0.40%, and 0.40%, respectively. These results can provide genomic resources for the breeding as well as speciation of *Clivia* species.

**Figure 1.**
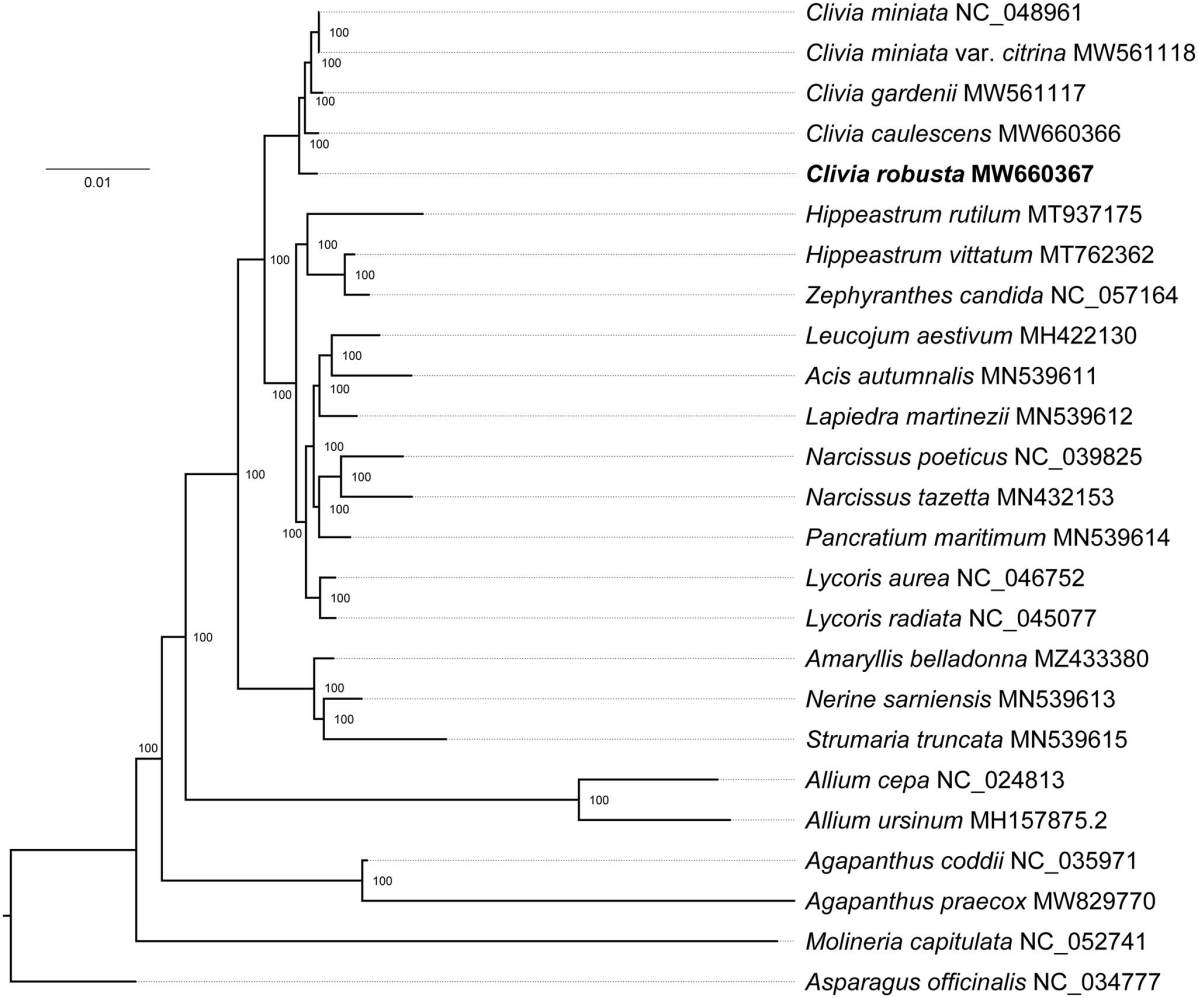
Maximum likelihood (ML) tree based on the complete chloroplast genome sequences of *C. robusta* and other 23 species, using *Asparagus officinalis* (Asparagaceae) as the outgroup. Bootstrap values are indicated at the nodes.

## Data Availability

The data that support the analysis and results of this study are openly available in Genbank with accession number (MW660367) (http://www.ncbi.nlm.nih.gov/). The associated BioProject, SRA, and BioSample numbers are PRJNA704719, SRR13781249, and SAMN18053628, respectively.
